# Multiple Primary Melanomas: Retrospective Review in a Tertiary Care Hospital

**DOI:** 10.3390/jcm11092355

**Published:** 2022-04-22

**Authors:** Rodolfo David Palacios-Diaz, Blanca de Unamuno-Bustos, Carlos Abril-Pérez, Mónica Pozuelo-Ruiz, Javier Sánchez-Arraez, Ignacio Torres-Navarro, Rafael Botella-Estrada

**Affiliations:** 1 Department of Dermatology, Hospital Universitari i Politècnic La Fe, 46026 Valencia, Spain; rodolfo.palaciosd@gmail.com (R.D.P.-D.); carlospeab@gmail.com (C.A.-P.); m.pozueloruiz@hotmail.com (M.P.-R.); jvsanchezarraez@gmail.com (J.S.-A.); ignaciotorresnavarro@gmail.com (I.T.-N.); rbotellaes@gmail.com (R.B.-E.); 2 Department of Medicine, Universitat de València, 46010 Valencia, Spain

**Keywords:** melanoma, multiple primary melanoma, frequency, regression, follow-up

## Abstract

Multiple primary melanomas (MPM) refer to the occurrence of more than one synchronous or metachronous melanoma in the same individual. The aim of this study was to identify the frequency of MPM and describe the clinical and histopathologic characteristics of patients with MPM. An observational single-center retrospective study was designed based on a cohort of melanoma patients followed in a tertiary care hospital. Fifty-eight (8.9%) patients developed MPM. Most patients were men (65.5%) and the median age at the time of diagnosis of the first melanoma was 71 years old. The median time of diagnosis of the second melanoma from the first melanoma was 10.9 months, and 77.6% of second melanomas were diagnosed within the first 5 years. In total, 29 (50%) and 28 (48.3%) first and second melanomas were located in the trunk, respectively. Concordance of anatomic site between primary and subsequent melanoma was found in 46.6% of the patients. Proportion of in situ melanomas was increasingly higher in subsequent melanomas (from 36.21% of first melanomas to 100% of fifth melanomas). An increasing rate of melanomas with histological regression was observed within subsequent melanomas (from 60.3% of first melanomas to 80% of third melanomas). Our results support the importance of careful long-term follow-up with total body examination in melanoma patients.

## 1. Introduction

Skin cancers are the most commonly diagnosed group of cancers worldwide [1]. A stable trend of rising incidence of cutaneous melanoma has been reported in the last decades. Melanoma incidence largely concentrates in highly developed countries, predominantly inhabited by people of European origin, with lighter skin and thus with a greater susceptibility to ultraviolet (UV) radiation [1]. Moreover, owing to its potential for metastasis, melanoma carries a high mortality burden.

Multiple primary melanomas (MPM) is a well-documented phenomenon that refers to the occurrence of more than one synchronous or metachronous melanoma in the same individual [2,3]. An expanding population at risk for a subsequent primary melanoma is the result of an increasing diagnosis of cutaneous melanoma and melanoma survival [4]. Previous studies have reported widely different frequencies of MPM, ranging from 1% to 13% [5]. This variability might be attributed to different populations, study design, and length of follow-up [4,5].

Risk factors for the development of a subsequent melanoma have been proposed in several studies. These factors include family history, personal history of dysplastic nevi, light color of hair, multiple common melanocytic nevi, and multiple cherry angiomas [6,7]. Histopathologically, the initial melanoma is usually the thickest, and subsequent melanomas are less invasive [3,4,6,7].

The present study aimed to identify the frequency of MPM in a tertiary care hospital from Spain. Moreover, we aimed to describe the clinical and histopathologic characteristics of the first and subsequent primary melanomas.

## 2. Materials and Methods

An observational single-center retrospective study was designed based on the information collected from a cohort of melanoma patients in the database of the Dermatology Department of Hospital Universitari i Politècnic La Fe, Valencia. Information was compiled from January 2014 to February 2022. All followed patients with cutaneous melanoma were eligible for analysis. The patients with more than one cutaneous melanoma, either in situ or invasive, were included.

Epidemiological, clinical, histopathological, and molecular variables were collected from the electronic medical records. Melanomas were classified into synchronous and metachronous regarding the difference in time of diagnosis of the second melanoma from the diagnosis of the first melanoma. Synchronous melanomas were defined as those diagnosed simultaneously or within the first three months after the diagnosis of the first melanoma. Metachronous melanomas were those diagnosed after the first three months [8,9,10].

We performed a descriptive analysis. Statistical analysis was carried out using Stata version 17.0 and Microsoft Excel. Quantitative variables were expressed as mean and standard deviation, or median and 25–75th percentiles, depending on the normality of distribution of the variable. The present study was approved by the Ethics Committee of Hospital Universitari i Politècnic La Fe.

## 3. Results

Information was obtained from 646 patients diagnosed with melanoma during the period of data collection. Among the reference population, 58 (8.9%) patients developed MPM. These 58 patients developed a total of 129 melanomas, corresponding to a mean of 2.22 melanomas per patient. Most patients developed two primary melanomas (48/58; 82.8%), eight patients developed three melanomas (13.8%), one patient developed four melanomas (1.7%), and one patient developed five melanomas (1.7%).

Table 1 describes epidemiological and clinical characteristics of patients with MPM. Epidemiological information was unavailable for one patient. Consequently, most analysis was done for 57 patients. Most patients (38/58; 65.5%) with MPM were men, and only 20 (34.5%) were women. The median age at the time of diagnosis of the first melanoma was 71 years old (range: 29 to 91 years old).

Patients had signs of chronic clinical actinic damage, identified by the presence of solar lentigines (46/57; 80.7%), actinic keratosis (21/57; 36.8%), and non-melanoma skin cancer (17/57; 29.8%). Furthermore, most patients had less than 50 nevi (48/57; 84.2%), and only four (7%) had previous history of histologic evidence of dysplastic nevus. Four patients (7%) had concomitant family history of melanoma, and 30 patients (53.6%) had family history of other malignancies. None of the patients had a genetic disorder related to DNA repair, immunosuppression, or history of giant congenital nevus.

**Figure 1:**
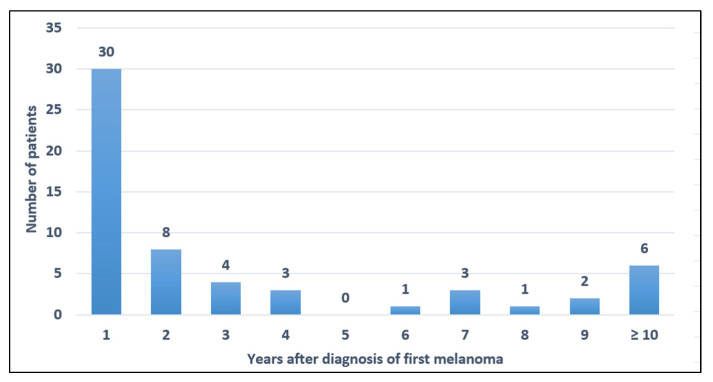
Time of diagnosis of the second melanoma from the first melanoma.

The median time of diagnosis of the second melanoma from the first melanoma was 10.9 months (range 0–196.67 months) (Figure 1). Twenty patients (34.5%) had synchronous melanomas, while 38 (65.5%) had metachronous melanomas. Most second primary melanomas (45/58; 77.6%) were diagnosed within the first 5 years of the first melanoma, and fifteen patients (25.9%) had a second primary melanoma diagnosed during the same medical appointment as the first primary melanoma. Nevertheless, six patients (10.3%) had a second primary melanoma after ten or more years of follow-up.

**Figure 2:**
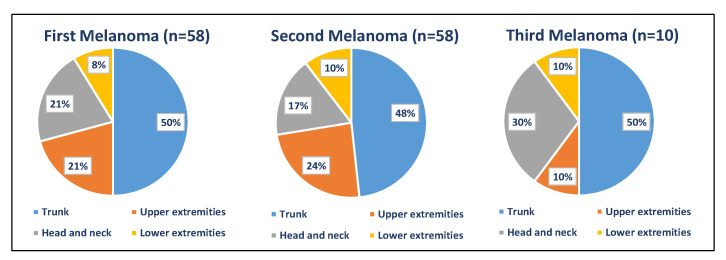
Anatomic location of first and subsequent primary melanomas.

The first primary melanomas were located mostly on the trunk (29/58, 50%), followed by the head and neck and the upper extremities (each one 12/58; 20.7%) (Table 2 and Figure 2). The trunk was also the most frequent anatomic site of second and third melanomas with 28/58 (48.3%) and 5/10 (50%), respectively. Site of the fourth melanoma was the head and neck (1; 50%) and lower extremities (1; 50%). The only patient that developed five melanomas had the latter melanoma located on the trunk. In 27 (46.6%) patients, second melanomas were located on the same anatomic region as first melanomas.

**Figure 3:**
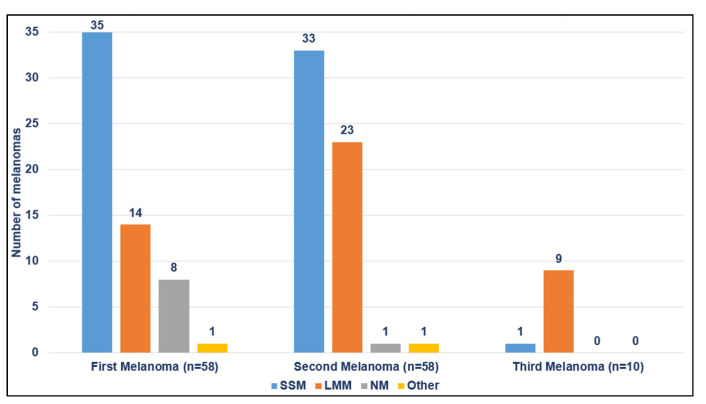
Histologic subtype of first and subsequent primary melanomas.

Regarding histopathological characteristics of MPM, superficial spreading melanoma (SSM) was the most common histologic subtype in first (35/58; 60.3%) and second (33/58; 56.9%) melanomas, while lentigo maligna (LM) histological subtype had the highest proportion in third melanomas (9/10; 90%) (Table 2 and Figure 3). An increasing rate of LM subtype within subsequent melanomas was observed. Moreover, 34 (58.6%) second melanomas had the same histologic subtype as the first melanoma.

**Figure 4:**
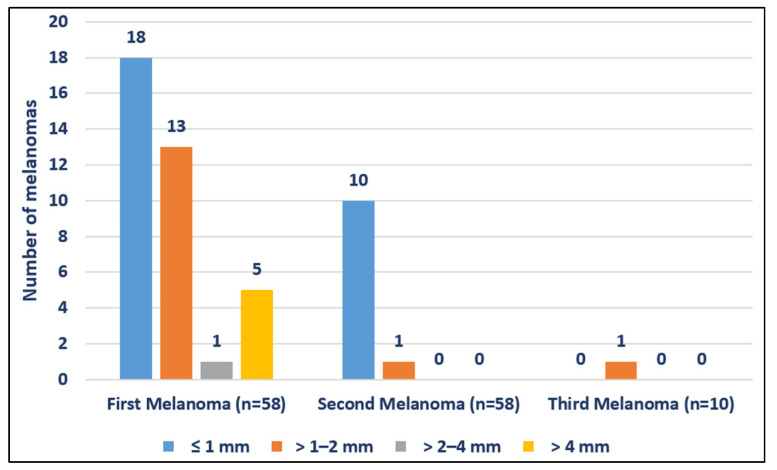
Breslow index of first and subsequent primary melanomas.

Patients with MPM developed less invasive subsequent melanomas. Twenty-one (36.2%) first melanomas were non-invasive (Table 2). Proportion of in situ melanomas was increasingly higher in second (46/57; 80.7%), third (9/10; 90%), fourth (2/2; 100%), and fifth (1/1; 100%) melanomas. Regarding Breslow thickness, invasive second melanomas were thinner than invasive first melanomas (mean Breslow thickness: 0.65 mm vs. 1.54 mm, respectively) (Figure 4). Only one (10%) of the third melanomas was invasive and had a Breslow thickness of 1.4 mm.

**Figure 5:**
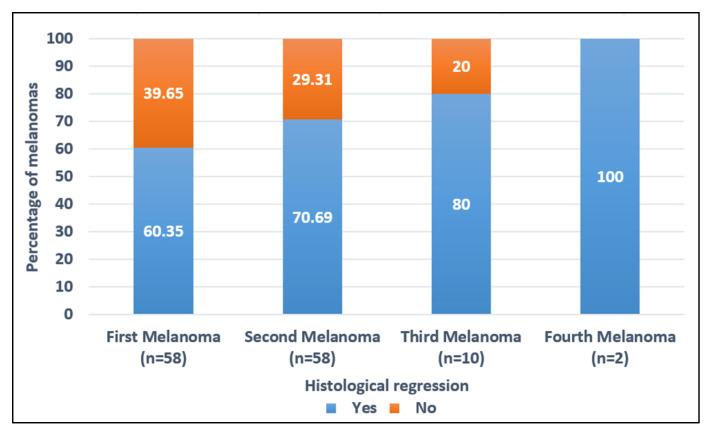
Rate of first and subsequent primary melanomas with histological regression.

An increasing rate of melanomas with histological regression within subsequent melanomas was observed. While 60.3% (35/58) of first primary melanomas showed regression, 70.7% (41/58) and 80% (8/10) of the second and third melanomas, respectively, had regression in histologic examination (Table 2 and Figure 5). Neural invasion or microscopic satellitosis was not found in any melanoma. Only one first melanoma had vascular invasion. An associated melanocytic nevus was reported in 18.9% (11/58) first primary melanomas, 31% (18/58) second primary melanomas, and 30% (3/10) third primary melanomas. The most frequent pre-existing lesion was common nevus.

Sentinel lymph node biopsy was performed in 18 (31%) first melanomas and in only 1 (1.7%) second melanoma. Only one sentinel lymph node biopsy of the first primary melanomas was positive. Regarding molecular data, mutations in the melanoma susceptibility gene, *CDKN2A*, were studied in only four (6.9%) patients, and only one carried the variant V59G of *CDKN2A*.

Among all the patients with MPM, only four (6.9%) experienced locoregional recurrence. These four patients had only two primary melanomas each. There was only one melanoma-related death in a patient with history of two primary invasive melanomas.

## 4. Discussion

Melanoma patients with either invasive or in situ cutaneous melanoma have an elevated risk for developing a subsequent primary melanoma [11,12]. Our results showed an approximately 9% rate of MPM among patients with cutaneous melanoma. Although this data is consistent with previous studies [5], a subsequent primary melanoma was a more common phenomenon than in several previously reported studies (Table 3) [6,7,8,9]. This variability may be explained by different methodological approaches or by an increase in the worldwide incidence of cutaneous melanoma in recent decades [5,13,14]. On the other hand, there is also growing evidence suggesting that overdiagnosis may play an important role in this trend, at least regarding thin lesions [14,15]. Increased diagnostic scrutiny, including more screening skin examinations, lower clinical threshold to biopsy a pigmented lesion, and lower pathological threshold as well as poor reproducibility criteria to label a lesion as melanoma, may explain the rising melanoma diagnoses to some extent [16,17].

According to previous reports, most subsequent primary melanomas (52%) were diagnosed within the first year from the time of the first melanoma diagnosis [5,6,7]. Furthermore, 25.9% of our patients had a second melanoma diagnosed in the same medical consultation as the first melanoma. This highlights the importance of performing a comprehensive skin examination during the initial as well as following visits in melanoma patients [5,8]. Additionally, 10.3% of our patients were found to have a subsequent primary melanoma after ten or more years of follow-up. The longest time to diagnosis of a second melanoma was 17 years. On this regard, McCaul et al. found an overall incidence of second primary melanoma in the first year of 12.7 per 1000 person-years and a constant 6.01 per 1000 person-years thereafter up to 20 years [18]. Avilés-Izquierdo et al. found a high proportion of self-detected primary cutaneous melanomas (69%) [19]. The patients with self-detected melanomas presented with thicker Breslow index than patients with melanomas that were detected by dermatologists [19]. Contrastingly, Brobeli et al. reported that 93% of the second primary melanomas were recognized and diagnosed by the attending physician [20]. These observations might suggest the need for long-term follow-up in melanoma patients to achieve early detection.

Risk factors of subsequent primary melanomas have been already analyzed. Known factors include occurrence of nonmelanoma skin cancer, a high count of large or small nevi, and actinic skin damage [21]. Data regarding pigmentation phenotypes such as hair color, skin phototype, and MC1R variants are controversial, as previously reported studies have failed to demonstrate a significant impact on multivariate analysis [7,21]. Other factors include high risk of CDKN2A mutations and a positive family history of melanoma [21]. Most of our patients showed signs of chronic sun damage, represented by the presence of solar lentigos (80.7%), actinic keratosis (36.8%), and non-melanoma skin cancer (29.8%).

In contrast, only few patients had more than 50 nevi (15.8%), and even fewer had a family history of melanoma (7%). Thus, multiple nevi count and family predisposition, which may result in young patients with MPM, were not representative in our case series [21]. Consistent with previous studies, most of our patients were older, with a median age of 71 years old [3,4,6,7,21].

Our results showed that the trunk was the most common site for first and subsequent melanomas, as reported in previous studies [6,7,8,9]. In contrast, other authors have found different more common anatomic sites, such as the head and neck and lower limbs [4,22]. As reported before, we found a concordance of location for the first and second melanomas in 46.6% patients of our cohort [3,8,9]. In this regard, our results may support the possible field effect of susceptibility reported by other authors [9,23].

In line with other studies, SSM was the most common histological subtype in first and second melanomas [7,8,9,22,24]. In addition, we found an increasing rate of LM histological subtype with subsequent melanomas, which may be related to the high degree of cumulative exposure to UV radiation in our patients. Consequently, active preventive measures against chronic sun damage should be stressed in patients with MPM. Regarding the presence of a pre-existing melanocytic lesion, previous studies have reported an overall rate of nevus-associated melanoma of 30% [25]. In our cohort, subsequent primary melanomas had a higher rate of associated nevus than the first melanomas. The rising proportion of underlying nevus might be the result of early detection of malignant changes due to close surveillance.

Moreover, as previously reported, invasive subsequent melanomas were thinner than the first invasive melanoma, and there was a rising rate of in situ melanoma in subsequent lesions. One possible explanation for this phenomenon is early detection due to close surveillance. This finding could emphasize the importance of adherence to a strict follow-up regimen to enable the identification of thinner melanomas [26]. As other authors have suggested, patient education combined with careful follow-up may have even more impact on early detection of subsequent melanomas [27]. Another hypothesis was that there is a different biological behavior in patients with MPM and single primary melanomas (SPM). In this regard, Summa et al. found that certain germline mutations, such as those of PIK3CA and CYP1B1, may contribute to development of MPM and SPM, respectively, suggesting different molecular developments [28]. Melanoma is an immunogenic tumor that may be affected by host immunity and regulated by genetic germline variations [29]. Ferguson et al. found association of genetic variants related to the expression of immunomodulatory genes with MPM. The most significant result was for rs2071304. Patients that carried the alternate allele G of rs2071304, which is associated with decreased expression of SPI1, were 40% less likely to develop MPM [29]. These findings suggest that immune modulation may be a contributing factor affecting the development of additional primary tumors in patients with SPM.

Strikingly, we found that subsequent melanomas had a higher rate of histological regression than first melanomas. Prognostic impact of regression has been controversial; however, recent evidence supports a more favorable prognosis in primary melanomas with histological regression [30,31,32,33]. In addition, Saleh et al. proposed regression as an immunologic surveillance response by antigen-specific cytotoxic lymphocytes following a “immunization effect” from being exposed to previous melanomas [34]. Similarly, Martin et al. found that regression was more frequent in second melanomas than in first melanomas [35]. Nevertheless, Zoller et al. found no statistically significant difference in regression in first and second primary melanomas [36]. Histological regression may also explain the lower Breslow thickness in subsequent melanomas due to the disappearance of melanoma cells by immune response in successive melanomas. Further investigations are needed to elucidate the role of the immune system and histological regression in patients with MPM.

The main strength of our study was the uniform and careful data collection in a single institution for a considerably long time. We reviewed clinical and histopathological charts to avoid missing data. Nevertheless, the retrospective and descriptive study design of our study prevented us from extracting analytic conclusions. Moreover, data were collected from patients of a single tertiary center. Consequently, our frequency might differ from frequencies reported in population-based studies. Furthermore, though data regarding many histopathological characteristics of interest were gathered, we lacked studies of genetic mutations for most of the patients. Future studies with a larger cohort of patient and molecular features may be of interest to further characterize the first and subsequent primary melanomas.

## 5. Conclusions

In summary, we reported one of the largest case series of MPM with an overall prevalence of 8.9%. Most patients were old men with signs of long-term exposure to UV and without other risk factors. Almost half of the second melanomas were located on the same anatomic region as first melanomas, although a significant number also appeared in other anatomical sites. This underscored the importance of total body examination during the first visits and in follow-up in melanoma patients. The occurrence of second melanomas after ten or more years might suggest the need for lifetime clinical follow-up. However, the fact that most second primary melanomas are in situ and the rising evidence suggesting melanoma overdiagnosis call for reassessment of the appropriate follow-up for melanoma survivors.

## Figures and Tables

**Table 1:** Epidemiological and clinical characteristics of patients with multiple primary melanomas.

	MPM (n = 58) n (%)
Women	20 (34.48%)
Age	
Mean (SD)	69.07 (14.48)
Median (25–75th percentile)	71 (63–80)
Fitzpatrick Skin phototype *	
I	3 (5.26%)
II	22 (38.60%)
III	28 (49.12%)
IV	4 (7.02%)
Severe sunburns *	34 (59.65%)
Chronic sun exposure *	8 (14.04%)
UVA rays exposure	4 (7.02%)
Freckling *	3 (5.26%)
Lentigines *	46 (80.70%)
Actinic keratosis *	21 (36.84%)
Non-skin cancer *	5 (8.77%)
Non-melanoma skin cancer *	17 (29.82%)
Congenital nevi *	4 (7.02%)
Common nevi *	
<50	48 (84.21%)
>50	9 (15.79%)
History of histologically confirmed dysplastic nevi *	4 (7.02%)
Family history of melanoma *	4 (7.02%)
Family history of non-melanoma cancer *	30 (53.57%)

* No available information for one patient; MPM: multiple primary melanomas; SD: standard deviation; UVA: ultraviolet A.

**Table 2:** Clinical and histopathological characteristics of multiple primary melanomas.

	First Melanoma n = 58, n (%)	Second Melanoma n = 58, n (%)	Third Melanoma n = 10, n (%)	Fourth Melanoma n = 2, n (%)	Fifth Melanoma n = 1, n (%)
Location					
Trunk	29 (50%)	28 (48.28%)	5 (50%)		1 (100%)
UE	12 (20.69%)	14 (24.14%)	1 (10%)	1 (50%)	
H&N	12 (20.69%)	10 (17.24%)	3 (30%)	1 (50%)	
LE	5 (8.62%)	6 (10.34%)	1 (10%)		
Histologic subtype					
SSM	35 (60.34%)	33 (56.90%)	1 (10%)	2 (100%)	
LMM	14 (24.14%)	23 (39.66%)	9 (90%)		1 (100%)
NM	8 (13.79%)	1 (1.72%)			
Other	1 (1.72%)	1 (1.72%)			
In Situ Melanoma	21/58 (36.21%)	46/57 (80.70%) *	9/10 (90%)	2 (100%)	1 (100%)
Breslow (mm)	(n = 37)	(n = 11)	(n = 1)		
Mean (SD)	1.54 (1.67)	0.65 (0.49)	1.4		
≤1 mm	18 (48.65%)	10 (90.91%)	0 (0%)		
>1–2 mm	13 (35.14%)	1 (9.09%)	1 (100%)		
>2–4 mm	1 (2.70%)	0 (0%)	0 (0%)		
>4 mm	5 (13.51%)	0 (0%)	0 (0%)		
Clark level	(n = 56)	(n = 56)			
I	21 (37.50%)	46 (82.14%)	9 (90%)	2 (100%)	1 (100%)
II	14 (25%)	6 (10.71%)	0 (0%)		
III	14 (25%)	2 (3.57%)	0 (0%)		
IV	7 (12.50%)	2 (3.57%)	1 (10%)		
Ulceration	8 (13.79%)	1 (1.72%)	1 (10%)	0 (0%)	0 (0%)
Lymphocyte infiltration					
Peritumoral	24 (41.38%)	18 (31.03%)	1 (10%)	1 (50%)	0 (0%)
Intratumoral	12 (20.69%)	12 (20.69%)	1 (10%)	1 (50%)	0 (0%)
Tumor mitotic rate (mitosis/mm^2^)					
Mean	1.32	0.81	1		
<1	22 (59.46%)	7 (63.64%)	0 (0%)		
≥1	15 (40.54%)	4 (36.36%)	1 (100%)		
Regression					
None	23 (39.65%)	17 (29.31%)	2 (20%)	0 (0%)	100 (100%)
<50%	28 (48.28%)	30 (51.72%)	4 (40%)	2 (100%)	0 (0%)
>50%	7 (12.07%)	11 (18.97%)	4 (40%)	0 (0%)	0 (0%)
Vascular invasion	1 (1.72%)	0 (0%)	0 (0%)	0 (0%)	0 (0%)
Underlying histologic lesion	11 (18.97%)	18 (31.03%)	3 (30%)	1 (50%)	0 (0%)
Common nevus	9 (15.52%)	17 (29.31%)	3 (30%)	1 (100%)	
Dysplastic nevus	2 (3.45%)	1 (1.72%)			
Sentinel lymph node biopsy					
Done	18 (31.03%)	1 (1.72%)	1 (10%)	0 (0%)	0 (0%)
Positive	1 (5.56%)	0 (0%)	0 (0%)		

* No available information for one patient; SSM: superficial spreading melanoma; LMM: lentigo maligna melanoma; NM: nodular melanoma; MPM: multiple primary melanoma; SD: standard deviation; UE: upper extremities; LE: lower extremities; and H&N: head and neck.

**Table 3:** Summary of previous studies.

**Authors**	**Year**	**MPM/Total** **n (%)**	**Age (Mean)**	**N° of Primary Tumors (n/MPM)**	**History of Dysplastic Nevi** **n (%)**	**Family History of Melanoma** **n (%)**	**Synchronous** **n (%)**	**Most Frequent Location**	
								**1° Melanoma**	**2° Melanoma**
Ferrone C et al. [3]	2005	385/4484 (8.6)	55	866 (2.3)	101 (41) ^c^	53 (20)	139 (36) ^f^	Trunk	Extremities ^g^
Moore M et al. [4]	2015	1122/16,570 (6.8)	64.4	NA	NA	NA	NA	H&N	H&N
Hwa C et al. [6]	2012	61/788 (7.7)	63.7	155 (2.5)	NA	13 (21)	NA	Trunk	Trunk
Ungureanu L et al. [8]	2021	26/699 (3.7)	55.3	59 (2.3)	NA	NA	13 (45.5)	Trunk	Trunk
Salgüero-Fernandez I et al. [9]	2021	31	67 ^b^	84 (2.7)	10 (31) ^d^	6 (19)	39%	Trunk	Trunk
Müller C et al. [21]	2019	299/1648 (18.1)	62	NA	NA	16 (15.4)	NA	NA	NA
Menzies S et al. [22]	2017	99/2057 (4.8) ^a^	66	114 (2.5)	NA	NA	NA	NA	NA
Palacios-Diaz R.D. et al.	2022	58/646 (8.9)	69.1	129 (2.2)	4 (7) ^e^	4 (7)	20 (34.5)	Trunk	Trunk
**Authors**		**In-Situ Melanoma; n (%)**		**Breslow Mean**		**Histological Subtype**		**Histological Regression Rate**	
		**1° Melanoma**	**2° Melanoma**	**1° Melanoma**	**2° Melanoma**	**1° Melanoma**	**2° Melanoma**	**1° Melanoma**	**2° Melanoma**
Ferrone C et al. [3]		76 (21)	186 (50)	1.2	0.4	NA	NA	NA	NA
Moore M et al. [4]		476 (42.4)	599 (53.4)	1.05	0.83	NA	NA	NA	NA
Hwa C et al. [6]		NA	NA	0.96	NA	SSM	NA	NA	NA
Ungureanu L et al. [8]		2 (7.7)	17 (51.5)	NA	NA	SSM	SSM	NA	NA
Salgüero-Fernandez I et al. [9]		39%	58%	0.8	0.47	SSM	SSM	32	32
Müller C et al. [21]		NA	NA	NA	NA	NA	NA	NA	NA
Menzies S et al. [22]		24%	52%	1.21	0.36	SSM	LM/LMM	26	17
Palacios-Diaz R.D. et al.		21 (36.2)	46 (80.7)	1.5	0.7	SSM	SSM	60.4	70.7

^a^ Although 99 patients with MPM were considered initially, the authors excluded 53 patients due to lack of essential clinical data. ^b^ Median age. ^c^ Clinically and histologically diagnosed. ^d^ Only clinically specified. ^e^ Only histologically diagnosed. ^f^ Within 30 days of first melanoma. ^g^ Aggregates upper and lower extremities.

## Data Availability

The data presented in this study are available on request from the corresponding author.
